# Extension of the Lecture Term by the Medical Colleges

**Published:** 1848-07

**Authors:** 


### EXTENSION OF THE LECTURE TERM BY THE MEDICAL COLLEGES.

It will be recollected, that at the Medical Convention which was held in Philadelphia last year, resolutions were passed requesting the Colleges to extend the term to *six months*, and pledging the support of the Convention to such schools as adopted that and other proposed improvements. At the meeting of the American Medical Association this year, in the City of Baltimore, the Committee on Medical Education in their report, which was adopted by the Association, recommended that the term should be *five months;* and the resolution pledging the support by the Association of such schools only as adopted the suggestions of the Association, was passed over as partaking too much of the character of a menace.

All must rejoice at the spirit which is manifested in these proceedings, so much more in accordance with the dignity of the body than the hasty resolves of the previous year.

By the advertisements of several of the schools, we perceive that the lectures of the next session will commence at an earlier day than on former occasions, although some of those in the larger cities, as well as country places, are not disposed to change. The Boston School, and the University of the City of New York, adhere to their present system of four months.

In the Jefferson Medical College of Philadelphia, it will be seen by an advertisement on another page, the term will commence on the sixteenth of October, instead of the first Monday in November, as heretofore, by which arrangement the term will be extended three weeks. Practical Anatomy, however, is taught during six months of the year, commencing on the first of October; and the Clinical Lectures are continued, with the exception of a few weeks during hot weather, all the year round, without any charge to the student—so that instruction in the practical branches is actually given in the institution throughout ten months of the year. Similar arrangements likewise exist at the University of Pennsylvania, in which the regular lectures will commence on the same day as in the Jefferson College.

